# Supplementary material for: Controlling the broadband enhanced light chirality with L-shaped dielectric metamaterials
Source: Nat Commun. 2024 May 4;15:3757. doi: 10.1038/s41467-024-48051-4 (PMC11069550; doi:10.1038/s41467-024-48051-4)
Supplement: Supplementary file 1 — Supplementary Information [file 41467_2024_48051_MOESM1_ESM.pdf]

Supplementary Material

Controlling the broadband enhanced light chirality with L-shaped dielectric metamaterials

Ufuk Kilic[1,#], Matthew Hilfiker[1,2], Shawn Wimer[1], Alexander Ruder[1], Eva Schubert[1], Mathias Schubert[1,3], and Christos Argyropoulos[4*]

[1]Department of Electrical and Computer Engineering, University of Nebraska-Lincoln, Lincoln, NE 68588, USA

[2]Onto Innovation Inc., Wilmington, MA 01887, USA

[3] Solid State Physics and NanoLund, Lund University, P.O. Box 118, 22100, Lund, Sweden

[4]Department of Electrical Engineering, The Pennsylvania State University, University Park, PA 16803, USA

[#]ufukkilic@unl.edu, *cfa5361@psu.edu

S1. Chiroptical Characterization

The optical manifestation of chirality, also known as circular dichroism (CD), is the differential absorption of left from right circularly polarized light. The chirality response extraction via commercially available CD spectrographs is a user-friendly simplistic experimental technique and usually provides accurate enough results for optically isotropic samples. However, for samples that possess optical anisotropies, including linear or circular birefringence and linear dichroism, this method to measure chirality leads to erroneous purely chirality values. It also does not have the ability to measure the effect of the reflected circular polarized radiation on the chirality [1]-[3]. However, measurements based on the generalized spectroscopic ellipsometry within Mueller matrix configuration [4] solve this problem due to the superior ability of this method to differentiate the circular dichroism from other irrelevant to chirality optical anisotropies. The incoming uncollimated incoherent but polarized beam and the out-going beam after its interaction with the sample are represented by the Stokes vectors S_{in} and S_{out}, respectively. Obtaining S_{out} and extracting the effect of the sample on the polarization state of incoming electromagnetic radiation necessitates constructing a relation between S_{in} and S_{out}

by applying Mueller calculus. The relation between S_{in} and S_{out} involves a 4x4 real valued transformation matrix, so-called Mueller matrix, that stores the information of the sample.

S1.1. Ellipsometric measurement analysis

Here, we report the details of our spectroscopic ellipsometry experimental chiroptical characterization technique. This technique was developed to accurately extract the spectrum of both circular dichroism and the Kuhn's dissymmetry factor shown in the main paper. The schematic representations of the experimental setups for both reflection and transmission mode measurements are shown in Figs. S1(a) and (b), respectively. As an example, the resulting spectral evolution of the 4x4 Mueller matrix elements in transmission and reflection modes for all-dielectric (Si) L-shaped metamaterial presented in our main paper with a thickness of $\approx$ 181 nm and rotation angle of $\approx 38^{\circ}$ are demonstrated in Figs. S2(a) and (b), respectively. It should be noted that the Mueller matrix representation of spectroscopic ellipsometry provides an accurate way to reveal various optical anisotropies, including circular dichroism (M_{14} and M_{41}), circular birefringence (M_{23} and M_{32}), horizontal linear dichroism (M_{12} and M_{21}), horizontal linear birefringence (M_{34} and M_{43}), 45° linear dichroism (M_{13} and M_{31}), and 45° linear birefringence (M_{24} and M_{42}). One can also obtain such optical anisotropy information by a non-depolarizing sample from the differential decomposition of Mueller matrix elements [2]. The proposed metamaterial design has large values in all off-diagonal elements, as can be seen in Figs. S2(a) and (b). Such behavior is the signature of bianisotropic material response. This demonstrates the necessity of the structures being analyzed using spectroscopic ellipsometry by using the Mueller matrix configuration as it is currently pursued in our work.

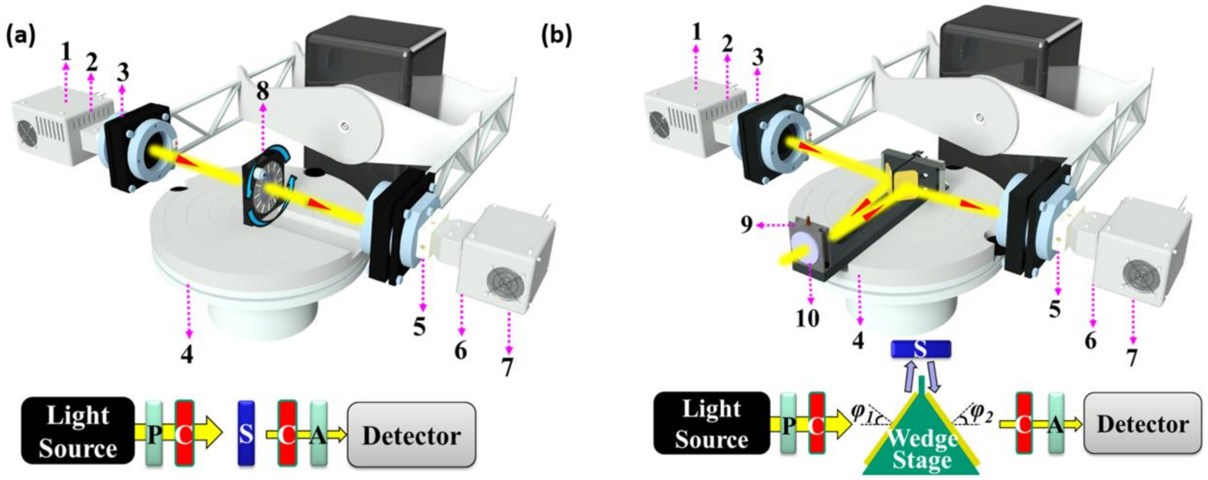

[1]:Light Source	[2]:Polarizer (P)	[3]&[5]:Compensator (C)	[4]:Main Stage
[6]:Analyzer (A)	[7]:Detector	[8]: Sample Stage with Rotation Ability	[9]:Sample (S)
[10]:Wedge Stage for Mirrors with vertical Tip-tilt Aligner			

Fig. S1: Experimental measurement setups to compute the g-factor spectra. Schematics of the spectroscopic ellipsometry-based optical setups for extracting the Mueller matrix elements in both (a) transmission and (b) reflection mode when operating at a normal angle of incidence. The optical components which were used are listed at the bottom table. Unlike the transmission setup, which employs a manual rotation stage for the sample, in the reflection mode a wedge stage with manual tip-tilt rotation ability is designed.

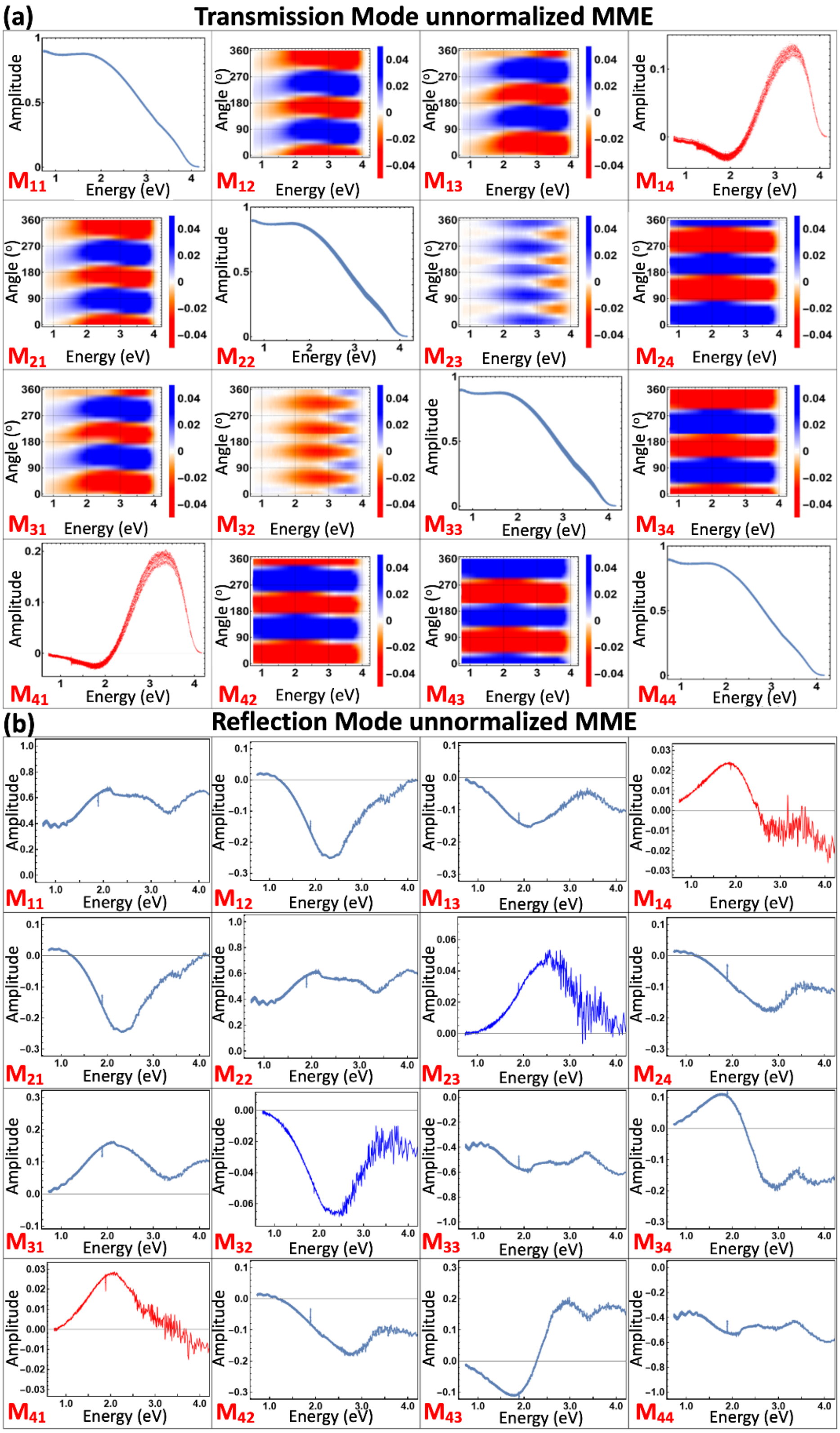

Fig. S2: Spectroscopic ellipsometry based measurement results. Mueller matrix spectra of all-dielectric Si L-shaped metamaterials at normal incidence in (a) transmission and (b) reflection modes by using the experimental setups shown in Figs. S1(a) and (b), respectively. While the transmission mode data acquisition is performed as a function of full azimuthal rotation angles ranging from 0° to 360° by 10° steps, the reflection mode data acquisition is performed at an arbitrary azimuthal orientation.

S1.2. Definition of circular polarized absorptance in terms of Mueller matrix elements

The generalized spectroscopic ellipsometry [4]-[7] measures the Mueller matrix elements [8], which represent the linear optical properties of any given sample under investigation. The Mueller matrix accurately describes the effect of the sample onto an incoming electromagnetic wave with any polarization. It relates the input Stokes vector with its outgoing Stokes vector:

$$\begin{pmatrix} S_0 \\ S_1 \\ S_2 \\ S_3 \end{pmatrix}_{output} = \begin{pmatrix} I_p + I_s \\ I_p - I_s \\ I_{45} - I_{-45} \\ I_+ - I_- \end{pmatrix}_{output} = \begin{pmatrix} M_{11} & M_{12} & M_{13} & M_{14} \\ M_{21} & M_{22} & M_{23} & M_{24} \\ M_{31} & M_{32} & M_{33} & M_{34} \\ M_{41} & M_{42} & M_{43} & M_{44} \end{pmatrix} \begin{pmatrix} S_0 \\ S_1 \\ S_2 \\ S_3 \end{pmatrix}_{input} . \quad \text{(S1)}$$

In the definition of Stokes vector components, the intensities: I_p, I_s, I_{45}, I_{-45}, I_+, and I_- are for the p-, s-, +45°, −45°, left-handed, and right-handed circularly polarized light components, respectively [4]. The dissymmetry factor (g_K) is computed by using the Mueller matrix elements measured at normal incidence in both transmission (T) and reflection (R) mode. By using the Mueller matrix shown in Eq. S1 [4]-[7] one can obtain the Stokes vector elements given by the formula: $S_{i-1}^{out,\pm} = \sum_{j=1}^{4} M_{ij} S_{i-1}^{in,\pm}$, where i=1,2,3, and 4 [9].

Note that the circular polarized radiation information is stored in the Stokes vector elements of both S_0 and S_3, as it is derived by Eq. S1. The other two Stokes vector elements (S_1 and S_2) are responsible for the linear polarized radiation that is assumed to be zero when circular polarized waves are solely used. Hence, the absorptance is written in terms of transmittance and reflectance for the LH and RH circular polarizations: $A_\pm = 1 - T_\pm - R_\pm$. Finally, the absorption-based Kuhn's g-factor definition is derived in terms of Mueller matrix elements and written as follows [1]:

$$g_K = 2\frac{A_- - A_+}{A_- + A_+} = 2\frac{(M_{14}^R + M_{41}^R) + (M_{14}^T + M_{41}^T)}{1 - M_{11}^R - M_{11}^T}. \quad \text{(S2)}$$

In our experimental setup, we employed a commercial spectroscopic ellipsometer with dual rotating compensators (J.A. Woollam Inc. RC2 ellipsometer model) that enables the collection of all 16 Mueller matrix elements. All the obtained results are normalized to the measurements

using the same setup but without the chiral sample in place. In the reflection mode, the extraction of circular polarized reflection performance depends on three factors. First, in addition to the actual chiral sample, there is a need for a calibration sample with known optical characteristics. Therefore, we fabricate a SiO_2 thin film ($t_{SiO2} \approx 489$ nm) on a low-doped (100) oriented silicon wafer by using the RF sputtering technique. Moreover, the incident light travels through Mirror-I (M1) - Sample (S) - Mirror-II (M2) system (see Fig. S1(b)). By using a DC magnetron sputtering system (ATC-2000F sputtering system purchased from AJA International), the mirrors are made up of gold thin films ($t_{Au} \approx 100$ nm) with ultrathin chromium (Cr) adhesion layers ($t_{Cr} \approx 10$ nm) deposited on low-doped (100) oriented Si wafers. It is therefore mandatory to account for the contribution of the mirror system on the outgoing beam in the detector side. Hence, the total measured reflected Mueller matrix is defined as follows:

$$\boldsymbol{M}^{R}_{Measured} = \boldsymbol{R}_4 \boldsymbol{M}_{Mirror_2} \boldsymbol{R}_3 \boldsymbol{M}^{R}_{Sample} \boldsymbol{R}_2 \boldsymbol{M}_{Mirror_1} \boldsymbol{R}_1, \tag{S3}$$

where $\mathbf{R_n}$ (n=1,2,3,4) is 4x4 coordinate rotation matrix that is given by:

$$\boldsymbol{R}_n = \begin{bmatrix} 1 & 0 & 0 & 0 \\ 0 & \mathrm{Cos}(2\theta_n) & \mathrm{Sin}(2\theta_n) & 0 \\ 0 & -\mathrm{Sin}(2\theta_n) & \mathrm{Cos}(2\theta_n) & 0 \\ 0 & 0 & 0 & 1 \end{bmatrix}, \tag{S4}$$

In Eq. S3, $\mathbf{R_1}$ and $\mathbf{R_4}$ account for the rotation of the first and second mirror planes relatively to the incident plane of the ellipsometer, while $\mathbf{R_2}$ and $\mathbf{R_3}$ are the minor rotation of the incident plane between the sample and each mirror. Therefore, the rotation angles are predicted as $\theta_1 = -\theta_4 \approx 90^\circ$ but $\theta_2 = \theta_3 \approx 0^\circ$. It is worth noting that all reflection mode measurements are performed with respect to a reference sample which is chosen to be a silicon wafer with a 2.5 nm native oxide layer. To account for the attenuation of Mueller matrix elements in the reflection mode, we performed a normalization protocol that was explained in our previous work [1]. Lastly, by using the spectroscopic ellipsometry data acquired within the spectral range from 0.72 eV to 6.4 eV at different angles of incidence ranging from 45° to 75° with 5° steps, the dielectric

functions of both the Au mirrors and SiO_2 thin film reference samples are accurately extracted. The extracted optical constants are also employed in the optical system model analysis.

S2. Transmission electron microscopy (TEM) imaging

As stated in the main paper, we employed a custom-built ultra-high vacuum glancing angle deposition (GLAD) instrument to bottom-up fabricate all-dielectric L-shaped metamaterials. It is worth mentioning that nanopillar-made thin films fabricated via the GLAD technique might exhibit imperfections in their structural parameters (especially pillar radius, slanting angles, etc.) which mainly stem from the anisotropic broadening effect, also known as the fanning phenomenon [10]. In this section, we demonstrate the main steps of the TEM imaging process that was used to perform the elaborate structural and elemental characterization of our metamaterial design. The resulting TEM sample preparation protocol is presented in Fig. S3.

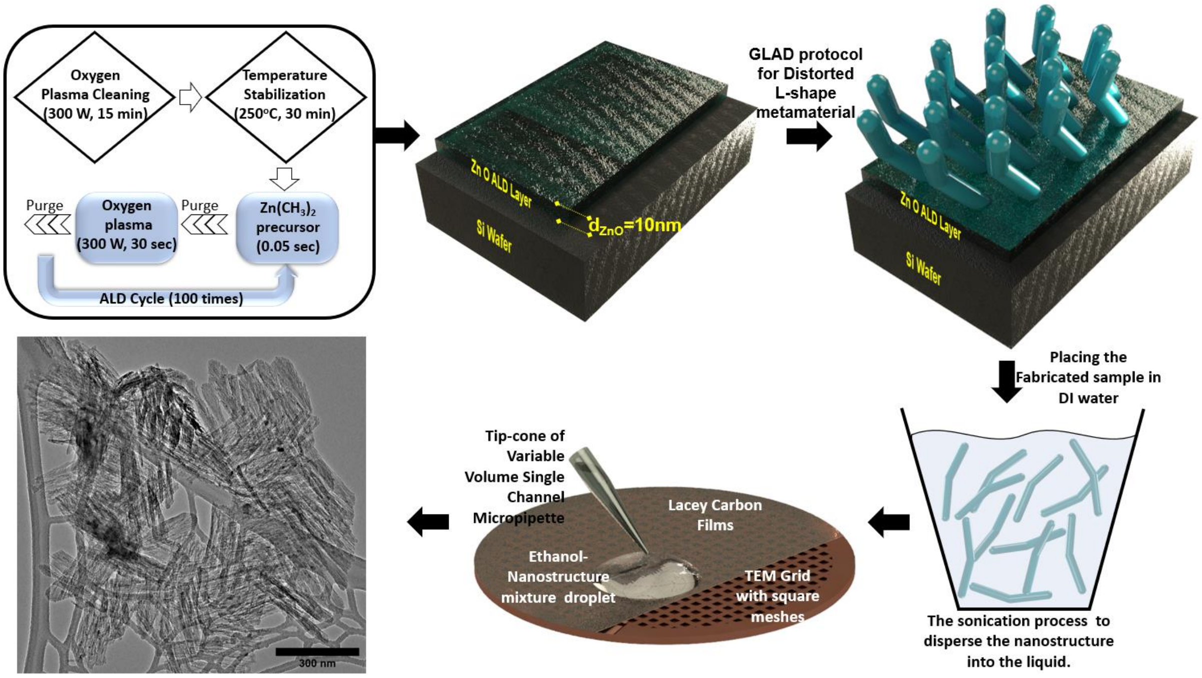

Fig. S3: Transferring the cluster of L-shaped nanorods to the liquid host matrix to perform TEM imaging. The process begins with ultrathin ZnO atomic layer deposited film that is soluble in DI water. The thickness of thin film is ≈10 nm. Following this process, the proposed nanostructure is assembled using the protocol shown in Fig. 1(b). Performing 5 minutes of sonication of the structures on ZnO flat thin film leads to the dispersion of structures in DI water. A high-resolution TEM image of isolated metamaterial structures is shown as an example.

A high-resolution STEM image of an isolated L-shaped nanostructure on TEM mesh grid stage is shown in Fig. S4(a). In addition to the structural parameters (e.g., thickness, slanting angle, pillar radius), the crystallographic state (see Fig. S4(b)) and the chemical composition (see Figs. S4(c) and (d)) of the fabricated nanostructures are investigated by using high-resolution scanning electron microscopy (SEM) (FEI Helios NanoLab 660 SEM instrument) and scanning (S)TEM with both high-angle annular dark-field (HAADF) and energy-dispersive X-ray (EDX) spectroscopy detector (FEI Tecnai Osiris S/TEM with Super-X EDX detector). The latter detector provides the ability to identify the elemental composition of the nanopillar. This is demonstrated in Fig. S4(d) where we image an isolated L-shaped metamaterial nanopillar. While copper (Cu) and carbon (C) peaks stem from the TEM copper grid system and carbon meshes, we also observe a Cloride (Cl) peak due to dispersion of the structures in water. Since we use ZnO ultrathin films as our sacrificial layer, we also observe a Zinc (Zn) peak in addition to Oxygen (O). We believe that the emergence of the Sulfide (S) peak is potentially a contamination effect in the solution or water. However, it is clearly demonstrated in Fig. S4(d) that the nanopillar is made of silicon (Si).

For the STEM imaging, a sacrifice layer on top of pristine low p-type doped Si wafer with (100) orientation is utilized. The sacrifice layer is a de-ionized-water-soluble ZnO ultrathin film which is fabricated by using an atomic layer deposition (ALD) system (Veeco CNT Fiji F200 ALD system). It is important to note that the proposed metamaterials are simultaneously fabricated on pristine Si wafer, glass, and Si wafer with ≈10 nm thick flat film of ZnO substrates. The fabrication of ZnO ALD employs $Zn(CH_3)_2$ as the main precursor (for 0.05 sec) and oxygen plasma as a co-reactant precursor (for 30 seconds). The main and co-reactant precursors are separated from each other by employing an argon purging process (for 30 sec). The substrate temperature is 250˚C. The 100-times repetition of the aforementioned recipe enables the fabrication of ≈10 nm thick ultrathin flat films. The thickness of ZnO ALD layer is obtained from the dynamic dual box model analysis [11] of *in-situ* spectroscopic ellipsometry data

acquired during the deposition of ZnO ultrathin film on the low p-type doped (100) oriented Si wafer. The details of the ALD recipe are given in Fig. S3. The resulting high resolution STEM images of the L-shaped metamaterial are shown in Fig. S4, where it is proven that the nanorods are mainly made of amorphous silicon.

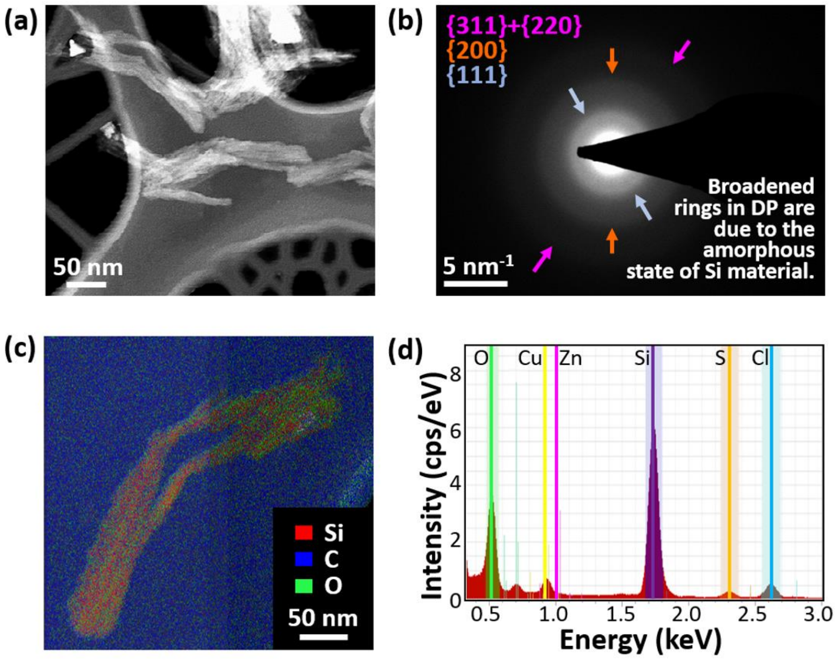

Fig. S4: Material analysis results of dispersed L-shaped nanorods. (a) STEM image of isolated L-shaped nanorods. (b) In order to investigate the structure's crystallography, the diffraction pattern (DP) is obtained. Broadened rings in DP are due to the amorphous state of Si material. (c) EDX spectrometry for selected material map over a HAADF image of an isolated structure on the TEM meshed grid stage. (d) The elemental composition of the nanorod obtained by using the EDX detector of the STEM instrument.

S3. Spectral versality factor comparison

As emphasized in the main paper, tailoring the spectral location of chirality extrema is the most nascent property of the presented L-shaped metamaterials. This section is devoted to performing a comparison in the spectral versality factor (χ) of the proposed chiral metamaterial platform and other metamaterials and metasurfaces presented in the literature that exhibit strong chirality. Table S1 lists their χ values which are experimentally extracted either from CD or g_K measured spectra.

Supplementary Table 1/Table S1

Spectral versatility factor comparison for different metamaterial designs

Structure Design	Varying Geometrical Parameter	χ Value	Chirality in terms of CD or g_K	Fabrication Method	Reference
**Plasmonic Hybrid Helical Metamaterial**	Helix	0.204	g_K	GLAD	[1]
**Planar Chiral Metasurface**	In-plane symmetry breaking distance (δ)	1.08	CD	e-beam Lithography	[12]
**Metal Nanocrescent Array**	Diameter	0.775	CD	Polystyrene microsphere template-based fabrication	[13]
**Helically Stacked Plasmonic Layers**	Polystyrene spheres diameter	1.07	CD	Dynamic shadowing growth	[14]
**Nanoscale Bouligand Multilayers**	Polystyrene spheres diameter	2.01	g_K	Grazing incidence spraying	[15]
**Nanohelix Metamaterial**	Total thickness	0.21	CD/g_K	GLAD	[16]
	Diameter	1.8	CD/g_K	GLAD	
**Core-Shell Helical Nanomaterials**	Shell Thickness	6.66	CD/g_K	GLAD and ALD	[17]
**L-shaped Metamaterial**	Total thickness	2.17	g_K	GLAD	this work
	Radius	9.84	g_K	GLAD	this work

S4. Bi-signate transmissive optical response

We observe the presence of both negative and positive values in the spectrum of experimentally obtained transmission-based g-factor values (g_T = 2($A_{T,LH}$ −$A_{T,RH}$)/($A_{T,LH}$ −$A_{T,RH}$) ≈ 2(T_{LH} −T_{RH})/(2−T_{LH} −T_{RH})) which are computed if we assume that the reflection of our samples is zero. Figure S5(a) shows the evolution of the g_T bi-signate response. Relevant simulations also verify this behavior, as depicted in Fig. S5(b). We believe that such behavior is remarkable due to its potential use in chiral emission applications where only the transmission of the photon emitter is required to be manipulated. The presence of bi-signate response will be a critical advantage because the proposed metamaterial can emit different handedness radiation at different parts of the spectrum.

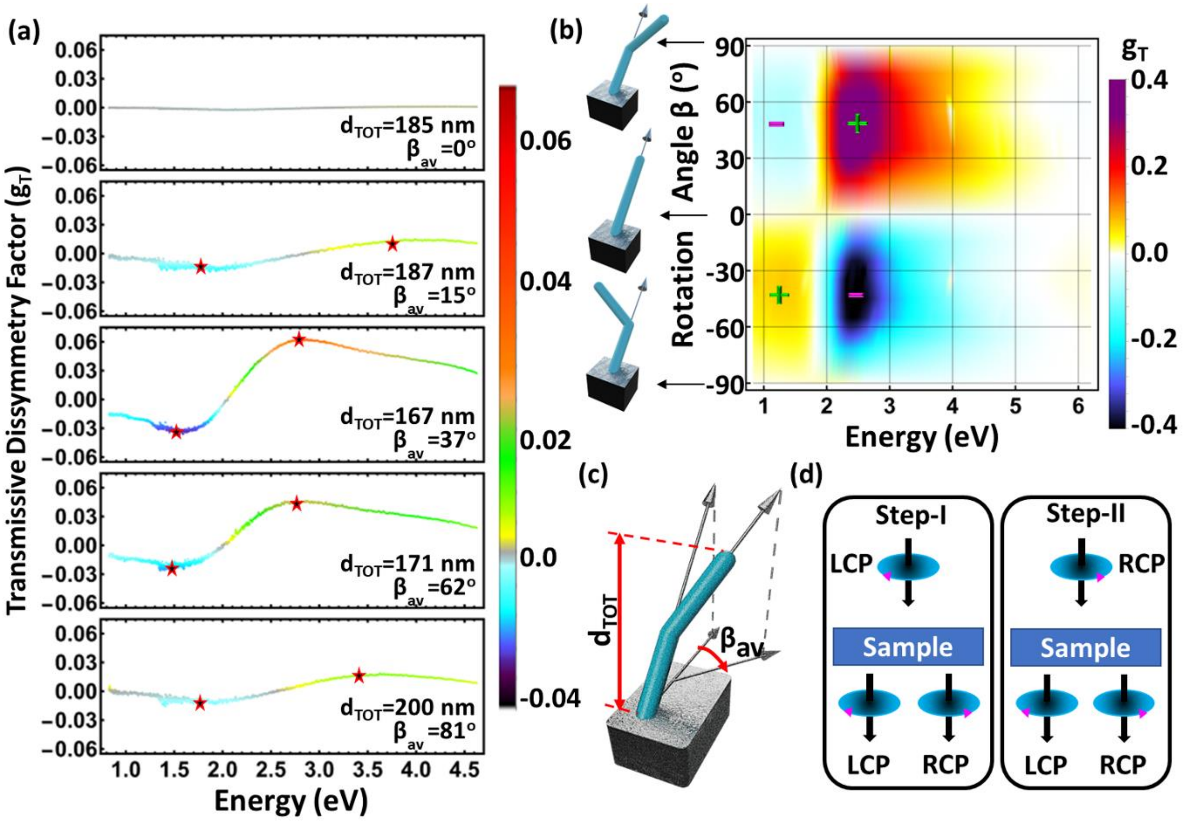

Fig. S5: Transmission based dissymmetry factor results. (a) Experimentally measured and (b) theoretically computed g_T spectra as a function of rotation angles. (c) Schematic of an isolated single L-shaped nanorod. (d) Schematic representation of the current transmission-based study. By exciting with LCP light, we measure the transmitted LCP and RCP response. We also perform the same measurement protocol when the incident light is RCP.

S5. Additional simulations

In this section, we present the results of additional simulations to further elucidate the enhanced and tailorable chirality response of our proposed new dielectric metamaterial structures. Our goal is to investigate the effects of several other L-shaped metamaterial structural parameters on the dissymmetry factor. Although it is beyond the scope of our current work, the presented varying structural parameters have realistic values and can be experimentally verified by using a seeding layer prior to the GLAD process. Figure S6(a) shows a 3D schematic representation of our metamaterial structure. Within this schematic, it is demonstrated that the nanopillars are arranged in hexagonal closed packing formation which is the closest approximation to the self-organized nanocolumnar growth mechanism of the GLAD process. Due to the nanocolumns slanting angle, the center-to-center adjacent distance between neighboring nanopillars along the x-direction is different from that along the y-direction (see the inset schematic in Fig. S6(a) of a cut-slice 2D view of the formation of the columns). The unit cell that is utilized in the simulations is depicted on a cross-section SEM image of our metamaterial design in Fig. S6(b). The green dashed lines in Fig. S6(b) are the boundaries of our unit cell which are chosen as a periodic boundary condition to account for an infinite number of nanopillars in our calculations. We present the color density plots of various Kuhn's dissymmetry factor spectra as functions of the change in the adjacent distance between neighboring pillars along both x (Δx) and y (Δy) directions (see Figs. S6(c) and (d), respectively), the slanting angle (θ_S, see Fig. S6(e)), and the pillar radius (R, see Fig. S6(f)). Interestingly, while the increase in both R and θ_S results in a red shift behavior in the main broad resonance, the change in the distance between neighboring pillars along x and y directions leads to a similar blue shift response. Additionally, we theoretically compute a record high spectral versatility factor of $\chi \approx 9.84$ corresponding to the change in the nanorod radius R of our proposed metamaterial design.

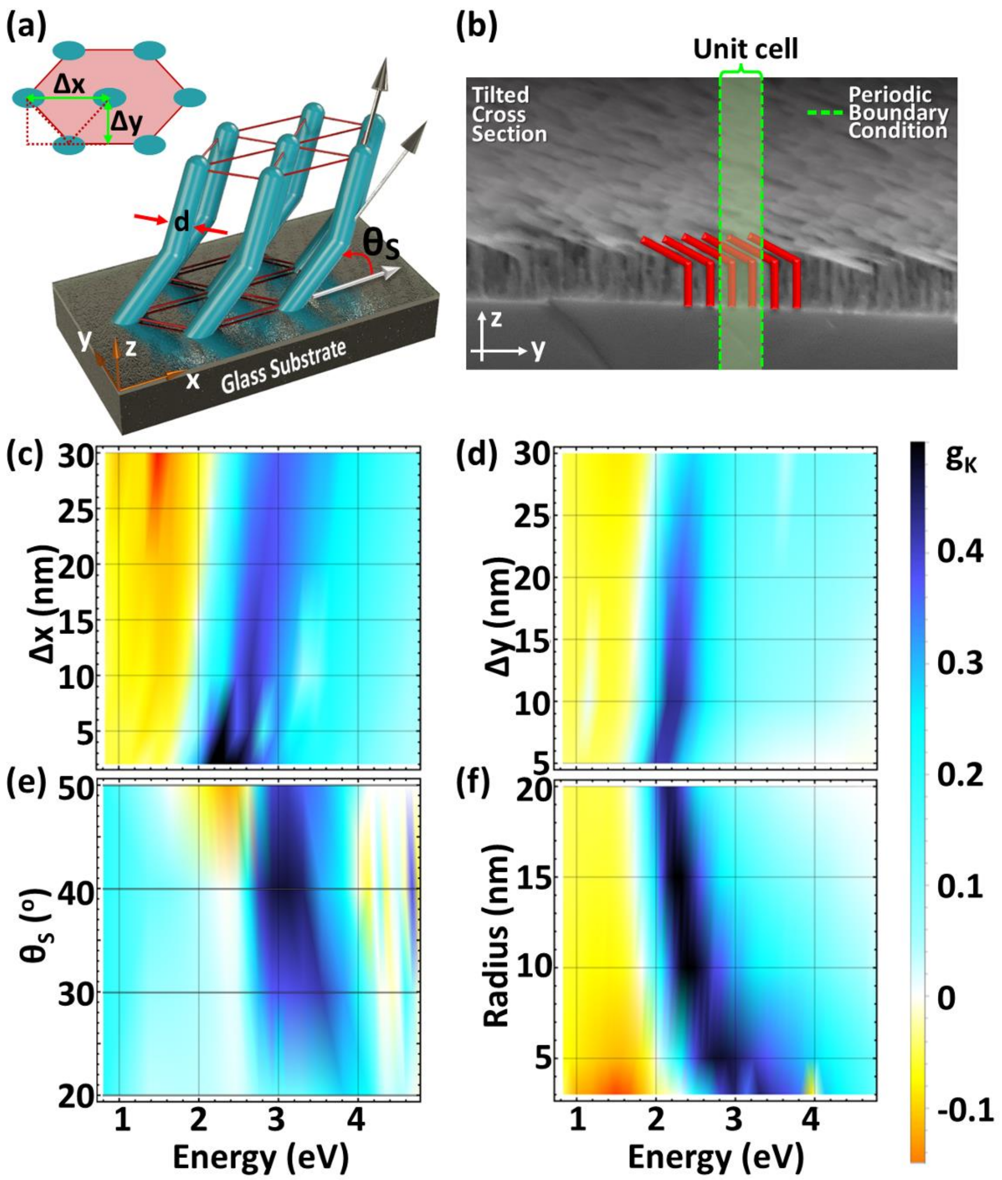

Fig. S6: Theoretical computation of g-factor spectra. (a) Schematic illustration of the nanorod array with a hexagonal formation. (b) SEM image of the proposed metamaterial where some nanorods are highlighted in red. The green dashed lines are used in the simulations as periodic boundary conditions. (c)-(f) Theoretically computed Kuhn's dissymmetry factors as a function of different geometrical parameters. Change in the adjacent distance between two neighboring L-shape nanorods along (c) x and (d) y directions, (e) slanting angle, and (f) radius of the L-shaped nanorod.

S6. Scattering decomposition definition

As it is shown in the main paper, the scattering coefficient can be decomposed into electro-magnetic multipoles, and their sum is equal to the total scattering coefficient. The expansion of total scattering coefficient is given by [18]:

$$S_{TOT,j} = \sum_{i,j} S_{i,j} = S_{ED,j} + S_{MD,j} + S_{EQ,j} + S_{MQ,j} \cdots \quad \text{(S5)}$$

where j is either LCP or RCP, and S_{ED} is electric dipole, S_{EQ} is electric quadrupole, S_{MD} is magnetic dipole, and S_{MQ} magnetic quadrupole scattering coefficients. If circularly polarized light interacts with a chiral medium, the scattering coefficient of the LCP incident light will be different than the RCP case. Hence, this difference between the total scattering coefficients results in a new metric of scattering chirality, also known as total scattering dichroism ($\Delta S_{TOT} = S_{TOT,LCP} - S_{TOT,RCP}$). This metric can also be used for each electromagnetic multipole, helping us to unravel the main driving mechanism of chiral light-matter interactions for our proposed metamaterial design.

The general relation of S_{TOT} is written as follows [18]-[19]:

$$S_{TOT} = \frac{k^4}{6\pi|\boldsymbol{E}_{inc}|^2\varepsilon_0^2}\left[\sum_{\alpha}\left(|p_\alpha|^2 + \frac{|m_\alpha|^2}{c}\right) + \frac{1}{120}\sum_{\alpha}\left(\left|kQ^e_{\alpha\beta}\right|^2 + \frac{\left|kQ^m_{\alpha\beta}\right|^2}{c}\right) + \cdots\right], \quad \text{(S6)}$$

where $|\boldsymbol{E}_{inc}|^2$is the electric field amplitude of the incident plane wave, k is the wavenumber, c is the speed of light, and p_α, m_α, $Q^e_{\alpha\beta}$, and $Q^m_{\alpha\beta}$ are the electric dipole, magnetic dipole, electric quadrupole, and magnetic quadrupole moments, respectively. The corresponding relation of each moment is given below:

Electric Dipole: $$p_\alpha = \frac{i}{w}\int d^3\boldsymbol{r}\left(J^w_\alpha j_0(kr) + \left(\frac{j_2(kr)}{2r^2}\right)(3(\boldsymbol{r}.\boldsymbol{J}_w)r_\alpha - r^2 J^w_\alpha)\right), \quad \text{(S7)}$$

Magnetic Dipole: $$m_\alpha = \frac{3}{2}\int d^3\boldsymbol{r}\left(\left(\frac{j_1(kr)}{kr}\right)(\boldsymbol{r}\times\boldsymbol{J}_w)_\alpha\right), \quad \text{(S8)}$$

Electric Quadrupole: $Q_{\alpha\beta}^{e} = \frac{3i}{w}\int d^3\boldsymbol{r}\left[\left(\left(r_\beta J_\alpha^w + r_\alpha J_\beta^w\right) - 2(\boldsymbol{r}.\boldsymbol{J_w})\delta_{\alpha\beta}\right)\left(\frac{j_1(kr)}{kr}\right) + \right.$ (S9)

$\left.\left(5r_\alpha r_\beta(\boldsymbol{r}.\boldsymbol{J_w}) + \left(r_\alpha J_\beta + r_\beta J_\alpha\right)r^2 - r^2(\boldsymbol{r}.\boldsymbol{J_w})\delta_{\alpha\beta}\right)\left(\frac{j_3(kr)}{2kr^3}\right)\right],$

Magnetic Quadrupole: $Q_{\alpha\beta}^{m} = 15\int d^3\boldsymbol{r}\left[\left(r_\alpha(\boldsymbol{r}\times\boldsymbol{J_w})_\beta + r_\beta(\boldsymbol{r}\times\boldsymbol{J_w})_\alpha\right)\left(\frac{j_2(kr)}{(kr)^2}\right)\right],$ (S10)

where (α,β) are *x*, *y*, and *z* coordinates, $\delta_{\alpha\beta}$ is the Kronecker delta that equals 0 if $\alpha \neq \beta$ and equals 1 if $\alpha = \beta$, r_α, r_β are equal to *x*, *y*, and *z* distances depending on the indices (α,β), *w* is the angular frequency, and *j* is the spherical Bessel function. The induced electric current density $\boldsymbol{J_w}(r)$ is equal to $-iw\varepsilon_0(\varepsilon_r - 1)\boldsymbol{E_w}(\mathbf{r})$, where $\boldsymbol{E_w}(\mathbf{r})$ is the electric field distribution, ε_0 is the permittivity of free space, and ε_r is the relative permittivity of the material. It is important to note that the above set of equations is valid for any wavelength and size of any arbitrary particle. The incorporation of the above equation set in our simulations made possible the extraction of each electromagnetic mode chiral scattering coefficient that is presented in Fig. 5 in the main paper.

S7. Azimuthal rotation dependent anisotropic metamaterial properties

Here, we present and discuss the chirality performance of our metamaterial design in terms of circular dichroism (CD) and other optical activity properties as a function of the sample's azimuthal rotation. As it is discussed in the main text of the manuscript, the linear and circular optical activity properties of the current metamaterial design have comparable amplitudes that can potentially contaminate its chiral response when commercially available circular dichroism measurement instruments are used that are usually based on Stokes polarimetry. Moreover, unlike our proposed Mueller matrix polarimetry chirality extraction that considers both transmission and reflection spectra, the commercial circular dichroism measurement instruments neglect the reflection contribution in the circular dichroism computation. The inability to differentiate linear anisotropic properties, including linear dichroism and

birefringence, from the circular dichroism signal leads to an inaccurate and not precise chirality characterization from commercially available CD instruments. Moreover, commercial CD-spectropolarimeters are designed to measure isotropic samples, such as chiral nanostructures randomly dispersed in liquid solutions. Thereby, such instruments are unsuitable for the precise and accurate quantitative assessment of thin film or metamaterial chiroptical responses which can have anisotropic properties.

In addition to the CD spectra, circular birefringence (CB), linear dichroism (LD), and linear birefringence (LB) spectra are extracted by using the Mueller matrix polarimetry. While M_{32} and M_{23} elements are directly related to the circular birefringence, the linear birefringence of the sample can be computed by M_{34} and M_{43} elements. The linear dichroism is equal to M_{12} and M_{21} elements. More details about the Mueller matrix elements are presented in section S1.2 and Eq. S1. Here, we use the differential Mueller matrix formalism, which was previously applied to different organic or inorganic thin films, and compute the anisotropic optical activity properties by the formula [20]-[21]:

$$\mathrm{M} = \ln\begin{pmatrix} 1 & \mathrm{m}_{12} & \mathrm{m}_{13} & \mathrm{m}_{14} \\ \mathrm{m}_{21} & \mathrm{m}_{22} & \mathrm{m}_{23} & \mathrm{m}_{24} \\ \mathrm{m}_{31} & \mathrm{m}_{32} & \mathrm{m}_{33} & \mathrm{m}_{34} \\ \mathrm{m}_{41} & \mathrm{m}_{42} & \mathrm{m}_{43} & \mathrm{m}_{44} \end{pmatrix} = \begin{pmatrix} -\kappa & -\mathrm{LD} & -\mathrm{LD}' & \mathrm{CD} \\ -\mathrm{LD} & -\kappa & \mathrm{CB} & \mathrm{LB}' \\ -\mathrm{LD}' & -\mathrm{CB} & -\kappa & -\mathrm{LB} \\ \mathrm{CD} & -\mathrm{LB}' & \mathrm{LB} & -\kappa \end{pmatrix}. \quad \text{(S11)}$$

The differential Mueller matrix M is obtained from taking the logarithm of each Mueller matrix element normalized to m_{11} and κ is the isotropic amplitude absorption. These properties are computed as a function of frequency (plotted in eV units) and in-plane (azimuthal) sample orientation, φ. The CD spectra can alternatively be represented using the ellipticity metric that is computed as follows [20]:

$$\theta_{\mathrm{ell}} = \tan^{-1}\left(\frac{e^{\mathrm{CD}}-1}{e^{\mathrm{CD}}+1}\right). \quad \text{(S12)}$$

To demonstrate the detrimental effect of other optical anisotropies on the chirality signal acquired by this type of commercial Stokes polarimetry-based instruments, we fabricate another

right-handed L-shaped metamaterial on a glass substrate (d_{tot}=120nm, $\beta = 45^o$, and total substrate area=0.6x0.6cm^2) so that the new sample can fit into the sample housing of the commercial UV-Vis CD spectropolarimeter (J-815, JASCO). We performed ellipticity measurements from 1.5 eV to 4.5 eV as a function of the sample's azimuthal rotation angle ranging from 0° to 360° with 15° step using both transmission-based Mueller matrix polarimetry method and commercial CD measurement instrument based on Stokes polarimetry. Here, we used only transmission in our Mueller matrix polarimetry measurements to achieve a fair comparison with the conventional CD spectropolarimeter that cannot measure the reflected signal from the metamaterial. The computed ellipticity plots are shown in Fig. S7(a) and S7(b) for Mueller matrix and Stokes polarimetry, respectively. The commercial Stokes polarimetry-based CD measurements in UV to near-IR range were performed using the JASCO J-815 instrument equipped with one photomultiplier tube detector and photo acoustic modulator. The used typical scanning parameters are: i) scanning speed 10 nm/min, ii) data interval 1 nm, iii) data pitch 0.5 nm, iv) digital integration time 0.25 s, and v) accumulation one. It is important to note that our Mueller matrix polarimetry-based CD measurements are performed by using two different methods: a) decomposition of Mueller matrix elements and b) direct Mueller matrix data analysis [20]-[21], where both methods were found to produce similar results.

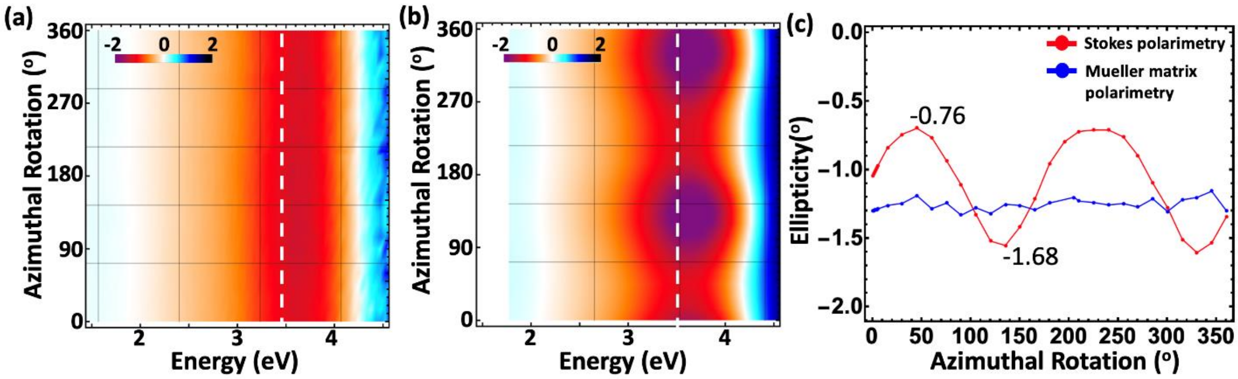

Fig. S7: Ellipticity spectra data. Measured ellipticity spectra as a function of azimuthal (i.e., in-plane) sample rotation obtained from (a) Mueller matrix polarimetry and (b) conventional Stokes polarimetry. (c) Ellipticity amplitude as a function of azimuthal rotation plotted at fixed 3.5 eV photon energy (white dashed lines in (a) and (b)).

While both Mueller matrix and Stokes polarimetry (commercially CD instrument) methods measure the same spectral location of ellipticity extrema and similar line shapes, the commercial instrument measures oscillations in the ellipticity spectra as a function of azimuthal rotation, which is erroneous result since the chirality or circular dichroism of the sample should not be anisotropic. The inaccurate chirality values oscillate with ~122% variation, as can be seen in Fig. S7(c) (red line). Chirality is expected to be independent from the azimuthal sample rotation, since it is an inherent symmetry breaking property of the structure, which results in its inability to be superimposed to its mirror image. This erroneous variation in the chirality strength is due to the inability of the commercial instrument to differentiate the purely chiral response from the other linear and circular optical activity anisotropies. Moreover, the average amplitude of ellipticity computed by conventional Stokes polarimetry is -1.25^{o} while the Mueller matrix polarimetry measures a constant value of -1.4^{o}, i.e., 0.25^{o} deviation exists from the correct result. Hence, the commercially available Stokes polarimetry method is unsuitable to extract the correct chirality values for transparent, birefringent, and dichroic 3D metamaterial samples.

Next, we further explore the optical anisotropic properties of the metamaterial sample, as was derived, and depicted in Fig. 5 in the main paper. A schematic on how the azimuthal sample rotation is achieved is demonstrated in Fig. S8(a). The metric of LD measures the differential attenuation between orthogonal linear polarization states, arising from either anisotropic absorption or scattering. The metric of LB characterizes the property causing a speed difference in linearly polarized light propagation along different orthogonal axes resulting in a phase difference. Using the transmission mode Mueller matrix polarimetry, the spectral evolutions of CD, CB, LD, LD at 45° polarization (LD’), LB, and LB at 45° polarization (LB’) as a function of azimuthal sample rotation are shown in Fig. S8. We observe that CB does not oscillate as a function of the sample’s azimuthal orientation, i.e., it is isotropic, similar to circular dichroism. However, the linear birefringence and dichroism are anisotropic, as was also shown in the main paper Fig. 5. Moreover, while the overall values of CB are comparable to CD, the other

anisotropic property terms have larger and always oscillating values (see Fig. S8). This explains the azimuthal angle-dependent CD signal values obtained from the conventional CD spectrometer (see Fig. S7b).

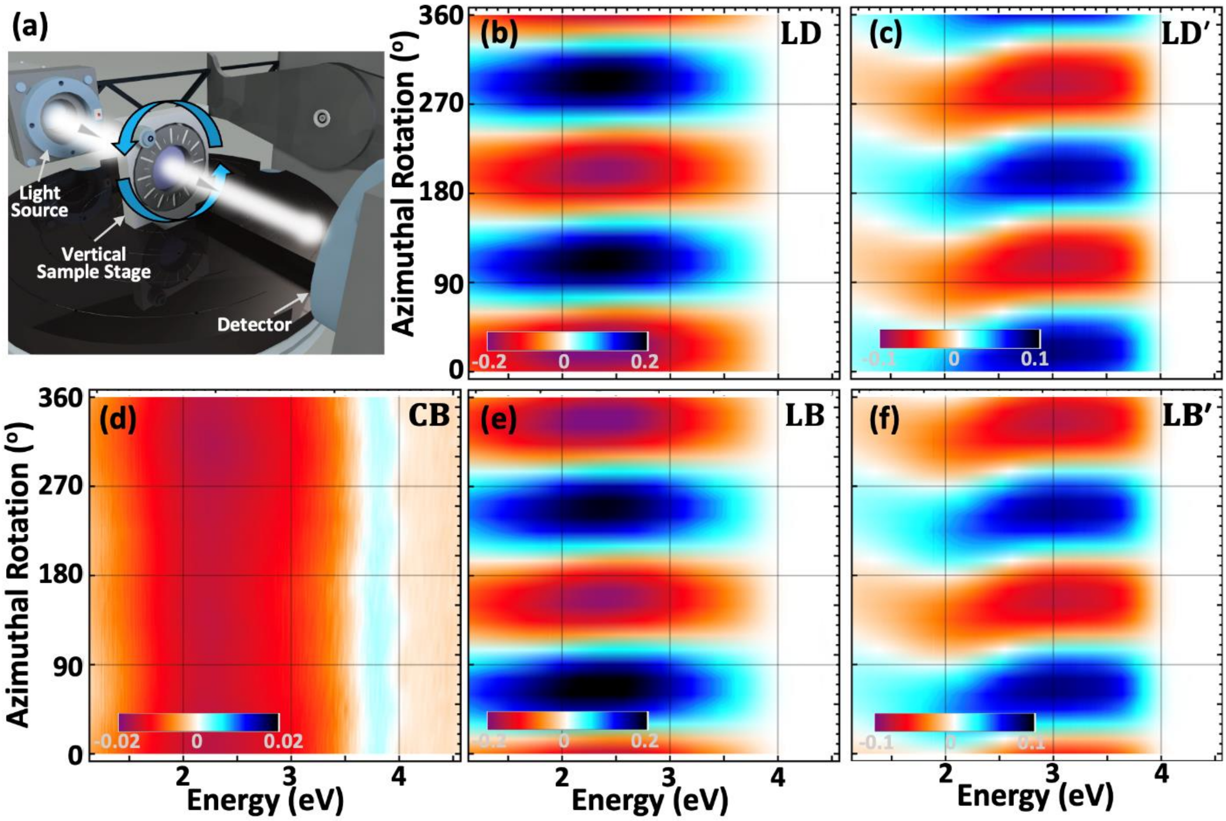

Fig. S8: Anisotropic optical properties of L-shaped metamaterials. (a) Schematic on how the azimuthal sample rotation is achieved. (b)-(f) Azimuthal rotation dependent spectra of (b) linear dichroism, (c) linear dichroism at 45° polarization, (d) circular birefringence (e) linear birefringence, (f) linear birefringence at 45° polarization.

S8. Amorphous state silicon optical constants

The anisotropic homogenization approach experimental extraction of the optical properties of amorphous Si is utilized [22]. The results are obtained by using reflection mode spectroscopic ellipsometry data analysis with a schematic shown in Fig. S9(a). The derived amorphous state Si optical constants are presented in Fig. S9(b), where the real (red line) and imaginary part (blue line/extinction coefficient) of refractive index are plotted as a function of the frequency. While the properties of amorphous silicon are computed by our in-house generalized spectroscopic ellipsometry measurements, the results agree very well with amorphous silicon

data provided in the literature [23]. The extracted optical constants are used in the simulations of our work.

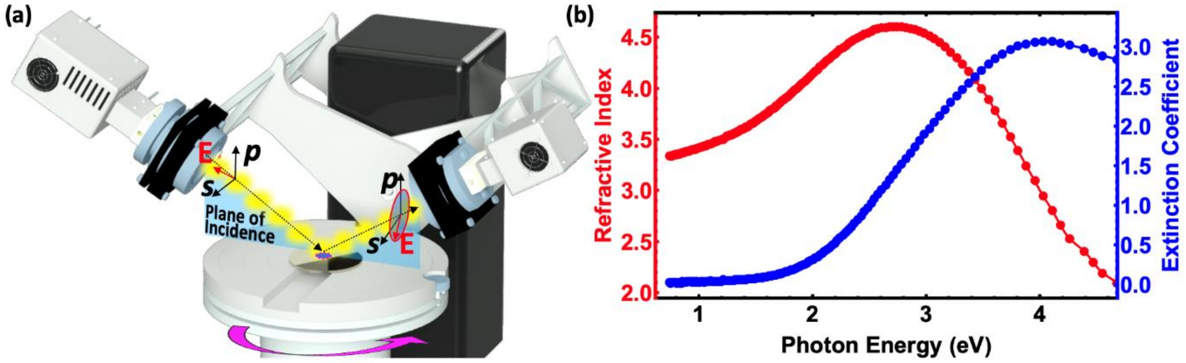

Fig. S9: Extracting the complex optical constant properties of amorphous silicon. (a) Reflection mode spectroscopic ellipsometry data analysis set-up. (b) Computed real and imaginary part of amorphous silicon refractive index spectra.

References

[1] Kilic, U., Hilfiker, M., Ruder, A., Feder, R., Schubert, E., Schubert, M. and Argyropoulos, C., *Broadband enhanced chirality with tunable response in hybrid plasmonic helical metamaterials*. Adv. Funct. Mater. 31, 2010329, **2021**.

[2] Schulz, M., Zablocki, J., Abdullaeva, O.S., Brück, S., Balzer, F., Lützen, A., Arteaga, O. and Schiek, M., *Giant intrinsic circular dichroism of prolinol-derived squaraine thin films.* Nat. Commun. 9, 2413, **2018**.

[3] Shindo, Y., Nakagawa, M. & Ohmi, Y. *On the problems of cd spectropolarimeters. ii: artifacts in cd spectrometers.* Appl. spectroscopy 39, 860–868, **1985**.

[4] Fujiwara, H. *Spectroscopic Ellipsometry* John Wiley & Sons, New York, **2007**.

[5] Schmidt, D. & Schubert, M. *Anisotropic bruggeman effective medium approaches for slanted columnar thin films*. J. Appl. Phys. 114, 083510, **2013**.

[6] Azzam, R. *Stokes-vector and Mueller-matrix polarimetry*. JOSA A 33, 1396–1408, **2016**.

[7] Hovenier, J. *Structure of a general pure Mueller matrix*. Appl. optics 33, 8318–8324, **1994**.

[8] Mueller, H. *Memorandum on the polarization optics of the photoelastic shutter*. Report of the OSRD project OEMsr-576 2, Massachusets Institute of Technology, **1943**.

[9] Delplancke, F. *Automated high-speed Mueller matrix scatterometer*. Appl. optics 36, 5388–5395, **1997**.

[10] Hawkeye, M. M. & Brett, M. J. *Glancing angle deposition: fabrication, properties, and applications of micro-and nanostructured thin films.* J. Vac. Sci. Technol. A . 25, 1317–1335, **2007**.

[11] Kilic, U., Mock, A., Sekora, D., Gilbert, S., Valloppilly, S., Melendez, G., Ianno, N., Langell, M., Schubert, E. and Schubert, M., *Precursor-surface interactions revealed during plasma-enhanced atomic layer deposition of metal oxide thin films by in-situ spectroscopic ellipsometry*. Sci. reports 10, 1–12, **2020**.

[12] Shi, T., Deng, Z.L., Geng, G., Zeng, X., Zeng, Y., Hu, G., Overvig, A., Li, J., Qiu, C.W., Alù, A. and Kivshar, Y.S., *Planar chiral metasurfaces with maximal and tunable chiroptical response driven by bound states in the continuum*. Nat. Commun. 13, 1–8, **2022**.

[13] Cao, L., Qi, J., Wu, Q., Li, Z., Wang, R., Chen, J., Lu, Y., Zhao, W., Yao, J., Yu, X. and Sun, Q., *Giant tunable circular dichroism of large-area extrinsic chiral metal nanocrescent arrays.* Nanoscale Res. Lett. 14, 1–7, **2019**.

[14] Larsen, G.K., He, Y., Ingram, W., LaPaquette, E.T., Wang, J. and Zhao, Y. *The fabrication of three-dimensional plasmonic chiral structures by dynamic shadowing growth.* Nanoscale 6, 9467–9476, **2014**.

[15] Hu, H., Sekar, S., Wu, W., Battie, Y., Lemaire, V., Arteaga, O., Poulikakos, L.V., Norris, D.J., Giessen, H., Decher, G. and Pauly, M., *Nanoscale Bouligand multilayers:*

Giant circular dichroism of helical assemblies of plasmonic 1D nano-objects. ACS nano, 15(8), pp.13653-13661, **2021**.

[16] Sarkar, S., Behunin, R. O. & Gibbs, J. G. *Shape-dependent, chiro-optical response of uv-active, nanohelix metamaterials*. Nano Lett., **2019**.

[17] Singh, H. J. & Ghosh, *A. Large and tunable chiro-optical response with all dielectric helical nanomaterials*. ACS Photonics 5, 1977–1985, **2018**.

[18] Alaee, R., Rockstuhl, C. & Fernandez-Corbaton, I. *An electromagnetic multipole expansion beyond the long-wavelength approximation*. Opt. Commun. 407, 17–21, **2018**.

[19] Jackson, J. D. *Classical electrodynamics*. John Wiley & Sons, **2021**.

[20] Schulz, M., Zablocki, J., Abdullaeva, O.S. et al. *Giant intrinsic circular dichroism of prolinol-derived squaraine thin films*. Nat. Commun. 9, 2413, **2018**.

[21] Arwin, H., Schoeche, S., Hilfiker, J., Hartveit, M., Järrendahl, K., Juárez-Rivera, O.R., Mendoza-Galván, A. and Magnusson, R., *Optical chirality determined from Mueller matrices. Appl. Sci.*, *11*(15), p.6742, **2021**.

[22] Kılıç, U., Mock, A., Feder, R., Sekora, D., Hilfiker, M., Korlacki, R., Schubert, E., Argyropoulos, C. and Schubert, M., *Tunable plasmonic resonances in Si-Au slanted columnar heterostructure thin films.* Sci. Rep. 9, 71, **2019**.

[23] D. T. Pierce and W. E. Spicer, *Electronic structure of amorphous Si from photoemission and optical studies*. Phys. Rev. B 5, 3017-3029, **1972**.
